# Endocrinology concepts for diabetic retinopathy care: bridging systemic metabolic control with retinal disease management and patient education

**DOI:** 10.3389/fmed.2026.1904880

**Published:** 2026-08-28

**Authors:** Sidney Jones, Peter Koulen

**Affiliations:** Vision Research Center, Department of Ophthalmology, School of Medicine, University of Missouri-Kansas City, Kansas City, MO, United States

**Keywords:** diabetes, diabetic retinopathy, metabolic disorders, retina, vascular disease, vision, visual function

## Abstract

Diabetic retinopathy (DR) is a leading cause of vision loss and a common microvascular complication of diabetes mellitus. Although diagnosis and management are typically guided by retinal findings, systemic metabolic factors—including glycemic control, diabetes duration, blood pressure, renal function, lipid status, and overall complication burden—play a central role in disease development and progression. Clinical decision-making often remains fragmented across endocrinology, primary care, and ophthalmology, contributing to variability in screening, referral, treatment selection, and patient education. This review proposes an integrated framework that applies endocrinology as a lens through which to better understand and manage diabetic retinopathy. The approach begins with a classification of systemic diabetes and metabolic disorders, then aligns these metabolically defined patient profiles with retinal diagnosis, monitoring, and stage-appropriate treatment strategies. Evidence is drawn from landmark endocrinology studies defining epidemiologic cohorts, diagnostic validation studies, and major randomized clinical trials as the basis for best practices in ophthalmological care. The goal is not only to synthesize evidence for clinicians, but also to support development of a concise patient-facing educational roadmap, presented as supplementary material, that summarizes disease progression, screening, treatment options, and anticipated next steps for patients. By integrating longitudinal endocrinology data with findings in ophthalmology, this framework may provide a strong rationale for interdisciplinary care, improve treatment outcomes, and promote more effective engagement of patients and their ophthalmologists in prevention and treatment of diabetic retinopathy.

## 1 Introduction

Diabetic retinopathy (DR) is a leading cause of preventable vision loss and one of the most clinically important microvascular complications of diabetes mellitus (4–8). Traditionally, DR has been conceptualized and managed as an ophthalmic disease, with diagnosis and treatment guided primarily by retinal imaging, severity staging, and the presence or absence of complications such as diabetic macular edema (DME), proliferative diabetic retinopathy (PDR), vitreous hemorrhage, or tractional retinal detachment (10, 13).

This retina-centered approach has enabled major advances in treatment, particularly through laser photocoagulation, anti-vascular endothelial growth factor (anti-VEGF) therapy, intravitreal pharmacotherapy, and vitrectomy (19–29). However, it does not fully account for the systemic nature of DR. Diabetic retinopathy represents a downstream manifestation of a chronic metabolic disease process. The retina is one site where cumulative glycemic exposure, vascular injury, inflammation, renal disease, blood pressure, lipid abnormalities, and broader diabetes complication burden become clinically visible (4–8, 10).

Mechanistic literature increasingly recognizes DR as a multifactorial disease involving microvascular dysfunction, inflammation, metabolic dysregulation, neuroglial injury, and retinal neurovascular unit disruption (10). This understanding is important because early retinal injury may precede clinically visible funduscopic changes (10). As a result, a purely image-based model may underrepresent early disease biology and may fail to incorporate the broader systemic trajectory that determines risk.

A substantial body of endocrinology literature has demonstrated that the development and progression of DR are strongly influenced by systemic factors, including glycemic control, duration of diabetes, blood pressure, lipid status, kidney disease, and overall metabolic phenotype. Landmark studies such as the Wisconsin Epidemiologic Study of Diabetic Retinopathy (WESDR, 4), the United Kingdom Prospective Diabetes Study (UKPDS, 5), the Action to Control Cardiovascular Risk in Diabetes Eye Study (ACCORD Eye, 6), and the Fenofibrate Intervention and Event Lowering in Diabetes (FIELD, 7) study demonstrate that systemic factors and systemic interventions can significantly modify retinal outcomes.

Despite this evidence, clinical decision-making in ophthalmology is often driven primarily by retinal findings at the point of examination. This may contribute to fragmented care between endocrinology, primary care, and ophthalmology. It also contributes to patient confusion: patients may know they have diabetes, but not understand how systemic control affects eye disease, why they need screening before symptoms appear, what each stage of DR means, or why one provider recommends observation while another recommends injections, laser, or surgery (19–25).

Accordingly, this review proposes that applying endocrinology as a lens to diabetic retinopathy provides a more comprehensive and clinically useful framework for understanding disease progression and guiding management (4–8, 16). The goal is to classify patients by systemic metabolic context first, then align that context with retinal diagnosis, risk stratification, treatment options, and patient-facing education (16, 26, 30). This approach is intended to support development of a concise patient education resource that translates the proposed endocrine-retina framework into language that is practical for patients and caregivers (4–8, 16, 26, 30).

Current clinical practice guidelines from the American Diabetes Association (ADA) and the American Academy of Ophthalmology (AAO) provide comprehensive evidence-based recommendations for diabetic retinopathy screening, diagnosis, and treatment (1, 31). These guidelines appropriately emphasize retinal disease severity, screening intervals, and therapeutic recommendations based on the best available clinical evidence. However, because they are organized primarily around ophthalmic disease stage and management decisions, they do not explicitly integrate the longitudinal systemic metabolic factors that shape retinal disease development and progression throughout the continuum of diabetes (1, 4–8, 10, 31).

The framework proposed in this review is intended to complement—not replace—existing clinical guidelines by reorganizing current evidence through an integrated endocrine-retina perspective. The proposed endocrine-retina framework is summarized in Table 1. Rather than beginning with retinal findings alone, this approach first considers the patient's systemic metabolic context, including diabetes duration, glycemic control, blood pressure, renal function, lipid status, and overall complication burden before linking these factors to retinal phenotype, diagnostic evaluation, stage-appropriate treatment, and patient education (4–10, 13–29). By emphasizing interdisciplinary communication between endocrinology, primary care, and ophthalmology, this framework seeks to provide a practical roadmap that supports clinical decision-making while helping patients understand how systemic metabolic health influences retinal disease progression and long-term visual outcomes (11, 12, 30, 32).

**Table 1:** Proposed endocrine–retina framework linking systemic metabolic disease to retinal phenotype and stage-specific management.

Endocrine context	Diagnostic criteria/systemic pattern	Likely retinal phenotype	Key evidence	Clinical question
Prediabetes/early diabetes	A1C 5.7–6.4% or early T2D; short duration; low burden	No DR or early/mild DR	ADA (1), DPP/DPPOS (2, 3), Abràmoff (11), Ipp (12)	How do we prevent and detect early disease?
Established diabetes with increasing risk	Longer duration; A1C above goal; comorbidities	Mild to moderate or moderate-severe NPDR	WESDR (4), Silva (14), PANORAMA (16), Protocol W (17)	When does monitoring become early treatment?
Edema-predominant disease	Longstanding diabetes; OCT-confirmed CI-DME	CI-DME with or without NPDR/PDR	DRCR OCT (15), Protocol T (18), VIVID/VISTA (19), Protocol V (20), YOSEMITE/RHINE (21), PHOTON (22)	When do we observe, inject, or reduce burden?
Proliferative phenotype	High microvascular burden; neovascularization	PDR/high-risk PDR	DRS (23), Protocol S (24, 25), CLARITY (26)	PRP vs anti-VEGF and how follow-up affects choice
Advanced complication phenotype	VH, TRD, non-clearing hemorrhage	Surgical-level disease	DRVS (27), Protocol N (28), Protocol AB (29)	Injection-first vs surgery-first

Proposed endocrine-retina framework integrating systemic metabolic disease with retinal phenotype, diagnostic evaluation, supporting evidence, and stage-specific clinical management. Unlike traditional clinical guidelines that are organized primarily by retinal disease severity, this framework begins with systemic metabolic risk and illustrates how endocrine disease progression informs ophthalmic diagnosis, treatment, and patient education. The framework is intended to complement existing ADA and AAO recommendations by emphasizing interdisciplinary care throughout the continuum of diabetic retinopathy.

## 2 Methods

A structured literature review was conducted to identify studies evaluating the relationship between systemic metabolic disease and diabetic retinopathy, as well as studies addressing retinal diagnosis, monitoring, and treatment. The work was designed as a structured narrative review with meta-analysis feasibility planning, rather than a completed quantitative meta-analysis at this stage.

Following peer review, an updated literature search was performed to incorporate recent evidence published through 2026, including advances in artificial intelligence, retinal neurodegeneration, electrophysiologic assessment, surgical outcomes, and contemporary diabetic retinopathy management.

### 2.1 Information sources

- PubMed/MEDLINE—Literature searches were conducted using PubMed/MEDLINE and Google Scholar databases. Searches focused on endocrinology and ophthalmology studies relevant to diabetic retinopathy screening, diagnosis, progression, systemic metabolic risk factors, and treatment strategies. Search terms included combinations of “diabetic retinopathy,” “diabetic macular edema,” “proliferative diabetic retinopathy,” “type 2 diabetes,” “prediabetes,” “anti-VEGF,” “screening,” “optical coherence tomography,” and “ultrawide-field imaging.” Priority was given to randomized controlled trials, landmark epidemiologic studies, prospective cohort studies, and clinically validated diagnostic studies.
- Google Scholar
- Journal publisher websites and trial network publication pages when needed for study identification and citation verification

### 2.2 Search terms

Search terms included combinations of the following: diabetic retinopathy, type 2 diabetes, prediabetes, A1C, glycemic control, diabetes duration, kidney disease, diabetic macular edema, center-involved diabetic macular edema, proliferative diabetic retinopathy, vitreous hemorrhage, anti-VEGF, aflibercept, ranibizumab, bevacizumab, panretinal photocoagulation, vitrectomy, diabetic retinopathy screening, artificial intelligence diabetic retinopathy detection, OCT diabetic macular edema, ultrawide-field imaging, diabetic retinopathy severity scale, diabetes subtypes, and Diabetes Complications Severity Index.

### 2.3 Study selection criteria

Priority was given to original studies, randomized clinical trials, landmark prospective epidemiologic studies, and diagnostic validation studies with standardized endpoints. Review articles and guidelines were used only for conceptual framing, clinical definitions, or background, not to identify underlying datasets as primary evidence.

**Inclusion criteria**

- Randomized clinical trials and large prospective studies involving DR, DME, PDR, or advanced DR complications
- Endocrinology studies with microvascular or retinopathy-related endpoints
- Landmark epidemiologic studies identifying systemic risk factors for DR development or progression
- Diagnostic, staging, screening, or imaging studies with validated endpoints
- Studies reporting measurable outcomes such as DRSS change, BCVA, OCT central subfield thickness, incidence of CI-DME/PDR, laser requirement, vitrectomy requirement, or treatment burden

**Exclusion criteria**

- Narrative reviews without primary data, except when used explicitly for scientific background
- Small case series without generalizable conclusions
- Studies lacking clinically interpretable outcomes relevant to endocrine-retina mapping

## 3 Results and discussion

### 3.1 Endocrine determinants of diabetic retinopathy development and progression

#### 3.1.1 Endocrine classification provides the foundation for risk assessment

##### 3.1.1.1 Goal

This section establishes the entry point of disease using standardized endocrine criteria rather than retinal findings. Patients are first classified according to diabetes and prediabetes definitions before being mapped to retinal outcomes. This section establishes that the review begins with systemic metabolic disease and then maps forward to retinal outcomes.

##### 3.1.1.2 Diagnostic criteria

- Prediabetes: A1C 5.7% to 6.4% or fasting plasma glucose 100 to 125 mg/dL (1).
- Diabetes: A1C 6.5% or higher or fasting plasma glucose 126 mg/dL or higher, when confirmed according to standard diagnostic criteria (1).

##### 3.1.1.3 ADA standards of care—diagnosis and classification of diabetes

The ADA Standards provide the formal endocrine definitions for diabetes and prediabetes. This source anchors the framework by ensuring that patient classification begins with measurable systemic criteria such as A1C, fasting glucose, and oral glucose tolerance testing rather than with retinal imaging alone. It is particularly important for the patient education goal because it allows the pamphlet to begin with categories patients already recognize: prediabetes, diabetes, and degree of metabolic risk (1, 4–8).

#### 3.1.2 Prevention of retinal disease begins before clinically apparent retinopathy

##### 3.1.2.1 Goal

This section examines whether early metabolic intervention may delay progression from prediabetes to overt diabetes and reduce downstream microvascular risk before clinically apparent retinal disease develops. Collectively, these studies support the concept that diabetic retinopathy prevention begins prior to the onset of visible retinal pathology through optimization of systemic metabolic control.

##### 3.1.2.2 ADA standards of care—prevention or delay of diabetes and associated comorbidities

This guideline section supports prevention as an active clinical strategy before retinal disease is present. It provides the evidence-based rationale for counseling patients with prediabetes and early type 2 diabetes about lifestyle intervention, weight management, and pharmacologic prevention when appropriate. In the manuscript, this paper helps justify including patients who have not yet developed clinical DR but are at systemic risk for future retinal disease (2, 4–8).

##### 3.1.2.3 Diabetes prevention program and diabetes prevention program outcomes study

The DPP and DPPOS demonstrated that lifestyle intervention and metformin reduced the incidence of diabetes over long-term follow-up (2, 3). The microvascular outcomes were nuanced, but participants who avoided progression to diabetes had lower prevalence of microvascular complications than those who developed diabetes (2, 3). For this review, the DPP/DPPOS supports the idea that patient education should begin before vision symptoms or retinal findings, while avoiding overclaiming that prediabetes treatment alone directly prevents all DR (2–8).

#### 3.1.3 Systemic metabolic risk factors drive retinopathy development and progression

##### 3.1.3.1 Goal

This section examines the systemic variables that influence diabetic retinopathy development and progression, including diabetes duration, cumulative glycemic exposure, blood pressure, kidney disease, dyslipidemia, and overall diabetes-related complication burden. Collectively, these factors support the concept that retinal disease progression reflects longitudinal systemic metabolic dysfunction rather than isolated ocular pathology.

##### 3.1.3.2 WESDR

WESDR is a foundational epidemiologic study showing that duration of diabetes and glycemic exposure are among the strongest predictors of retinopathy development and progression (4). Its importance for this framework is that it shifts interpretation away from a single retinal snapshot and toward a longitudinal disease model (4). It supports the review premise that DR risk begins in endocrine records and cumulative systemic disease trajectory before it becomes visible in the eye clinic (4, 8).

##### 3.1.3.3 Modern risk-factor studies

Contemporary risk-factor studies continue to support associations between DR/DME risk and systemic variables such as HbA1c, diabetes duration, systolic blood pressure, kidney disease, albuminuria, and treatment intensity (4–8). These studies strengthen the rationale for including metabolic comorbidity burden in retinal risk stratification. They also help move the review toward a more practical clinical model, where patients with similar retinal imaging may still have different predicted progression risk based on systemic features (4–8, 26, 30).

Emerging evidence further suggests that the effects of systemic metabolic dysfunction extend beyond microvascular injury alone. Chronic hyperglycemia promotes oxidative stress, activation of inflammatory cytokines, microglial activation, and disruption of the retinal neurovascular unit, contributing to retinal neuronal dysfunction before clinically apparent vascular abnormalities become visible. Increasing evidence indicates that inflammation and neurodegeneration develop in parallel during the early stages of diabetic retinopathy, with each process amplifying the other and accelerating retinal injury. This evolving understanding supports the concept that diabetic retinopathy should be viewed as a progressive manifestation of systemic metabolic disease rather than solely a vascular complication. Within the proposed endocrine-retina framework, these findings further strengthen the rationale for early optimization of glycemic control and other systemic risk factors before irreversible structural retinal changes occur (4–10, 33).

##### 3.1.3.4 Diabetes complications severity index

The Diabetes Complications Severity Index (DCSI) provides a quantitative method to summarize systemic diabetes-related complication burden across organ systems (8). Although it is not a retina treatment trial, it is highly relevant because it operationalizes the concept of whole-patient diabetes severity. In this framework, DCSI supports the argument that retinopathy should be interpreted in the context of systemic disease burden rather than treated as an isolated ophthalmic finding (8, 10).

#### 3.1.4 Systemic diabetes management influences retinal outcomes

##### 3.1.4.1 Goal

This section demonstrates that retinal outcomes can be modified through systemic endocrine management, thereby creating a direct mechanistic and clinical bridge between endocrinology and ophthalmology. Collectively, these studies support the concept that optimization of systemic metabolic control influences retinal disease progression, treatment burden, and long-term visual outcomes.

##### 3.1.4.2 UKPDS legacy follow-up

UKPDS demonstrated that early intensive glycemic control in newly diagnosed type 2 diabetes produced durable reductions in microvascular complications (5). Its relevance to this project is the concept of metabolic memory: early systemic control can influence later complication risk. This supports the patient-facing message that diabetes control matters even before eye symptoms begin (5).

##### 3.1.4.3 ACCORD eye

ACCORD Eye directly links systemic treatment to retinal outcomes by showing that intensive glycemic control and fenofibrate therapy reduced progression of diabetic retinopathy using standardized photographic grading (6). It is one of the most important bridge studies in the review because it uses endocrine interventions and ophthalmic endpoints. This makes it central to the argument that retina outcomes can be modified before the patient reaches treatment with injections, laser, or surgery (6, 18–29).

##### 3.1.4.4 FIELD

FIELD demonstrated that fenofibrate reduced the need for laser treatment for diabetic retinopathy in patients with type 2 diabetes (7). This is valuable because laser requirement is a clinically tangible ophthalmic endpoint, not merely a laboratory or risk-factor surrogate. It broadens the prevention message beyond glucose alone and supports the role of lipid-related pathways and systemic therapy in retinal outcomes (6, 7).

#### 3.1.5 Diabetes heterogeneity May influence retinal disease trajectory

##### 3.1.5.1 Goal

This section supports the concept that diabetes is not a uniform disease entity and that distinct metabolic phenotypes may influence complication risk, retinal progression, and long-term treatment requirements. Collectively, these studies suggest that patients with similar retinal findings may still demonstrate substantially different systemic disease trajectories and ophthalmologic outcomes based on underlying metabolic subtype characteristics.

##### 3.1.5.2 Bando et al. — Developing research for five subtypes of diabetes with specific characteristics

Ahlqvist et al. demonstrated through cluster-based analyses that diabetes is not a uniform disease but instead consists of distinct metabolic phenotypes with differing risks for complications (9). These analyses identified subgroups of adult-onset diabetes characterized by varying degrees of insulin resistance, insulin deficiency, obesity, and age at onset, each associated with differing risks for microvascular complications, including diabetic retinopathy (9).

Bando et al. subsequently expanded upon these observations by describing clinically relevant subtype characteristics and their potential implications for disease progression and management (30). These frameworks emphasize that patients with similar retinal findings may have fundamentally different underlying metabolic drivers. While such subtype classifications are not yet routinely implemented in clinical practice, they support the use of endocrinology as a lens for understanding variability in retinal disease and may contribute to more individualized risk stratification and treatment planning (9, 30).

### 3.2 Linking systemic disease stage to retinal phenotype and treatment

The following sections organize the literature by endocrine-defined patient context and then align each group with retinal phenotype, diagnostic approach, and treatment evidence. This structure keeps the patient as the unit of analysis rather than the eye alone.

#### 3.2.1 Group 1—Prediabetes and early diabetes: entry Into the retinal risk pathway

##### 3.2.1.1 Goal

This section focuses on screening, prevention, and early patient education in patients with prediabetes or early diabetes who may not yet demonstrate clinically apparent diabetic retinopathy. Although retinal findings may be absent at this stage, these patients have already entered the systemic metabolic risk pathway associated with future retinal injury and progression of diabetic eye disease (1–8).

##### 3.2.1.2 Diagnostic criteria

- Prediabetes by ADA criteria: A1C 5.7% to 6.4% or fasting glucose 100 to 125 mg/dL (1).
- Early type 2 diabetes: newly diagnosed or short duration, often less than 5 years (1, 4, 5).
- A1C near goal, low treatment intensity, and minimal known end-organ disease may indicate lower short-term risk, while rising A1C or comorbidities suggest greater concern (4–8).

##### 3.2.1.3 Abràmoff et al. — Pivotal trial of an autonomous AI-based diagnostic system for detection of diabetic retinopathy in primary care offices

Abràmoff and colleagues evaluated an autonomous AI-based diagnostic system for detection of diabetic retinopathy in primary care offices (11). The study is important because it shows how retinal screening can be moved closer to endocrine and primary-care workflows, reducing dependence on specialty access alone and serves as a practical tool for bringing endocrine-defined risk groups into the retinal care pathway before symptoms develop (11).

##### 3.2.1.4 Ipp et al. — Pivotal evaluation of an artificial intelligence system for autonomous detection of referrable and vision-threatening diabetic retinopathy

Ipp and colleagues evaluated an AI system for autonomous detection of referrable and vision-threatening DR in a multicenter study (12). This landmark study helped standardize referral thresholds and supports scalable detection outside traditional ophthalmology settings by enabling retinal screening to occur within primary care clinics, endocrinology practices, and other non-ophthalmic healthcare environments where patients with diabetes are routinely managed before referral to retina specialists becomes necessary (11, 12). This study reemphasizes the importance of screening modalities that can identify disease before a patient notices vision changes (11, 12).

##### 3.2.1.5 Conclusions

Early disease is often asymptomatic but detectable. The main intervention is systemic risk reduction plus reliable screening, not ocular treatment.

#### 3.2.2 Group 2—established diabetes: transition from systemic risk to clinically significant retinopathy

##### 3.2.2.1 Goal

This section examines patients with established diabetes who demonstrate increasing systemic metabolic risk and are beginning to develop clinically meaningful retinal pathology. At this stage, cumulative glycemic burden, diabetes duration, hypertension, renal disease, dyslipidemia, and overall complication severity increasingly correlate with progression from minimal retinal findings to clinically significant non-proliferative diabetic retinopathy, raising important questions regarding monitoring frequency, risk stratification, and the potential role of early retinal intervention (4–8, 13, 14, 16, 17).

##### 3.2.2.2 Diagnostic criteria

- Diabetes duration commonly 5 to 10 years or longer, though risk varies by control and phenotype (4–7).
- A1C persistently above individualized goal, often greater than 7% to 9% in higher-risk patients (1, 4–7).
- Increasing comorbidity burden such as hypertension, CKD, albuminuria, dyslipidemia, or insulin-requiring disease (4–8).
- Retinal phenotype: mild to moderate NPDR, or moderate-severe NPDR in the treatment-trial population (13, 14, 16, 17).

##### 3.2.2.3 Silva et al. — Peripheral lesions identified by ultrawide-field imaging predict increased risk of diabetic retinopathy progression

Silva and colleagues demonstrated that predominantly peripheral lesions identified on ultrawide-field imaging predict increased risk of diabetic retinopathy progression over four years (14). This study serves as an important bridge between systemic risk stratification and retinal phenotype because it demonstrates that conventional central retinal imaging may underestimate disease burden and progression risk in certain patients. It further supports the concept that improved retinal staging may help refine monitoring intensity, identify higher-risk patients earlier in the disease course, and better define the transition toward treatment consideration (14).

##### 3.2.2.4 PANORAMA

PANORAMA evaluated aflibercept in patients with moderately severe to severe NPDR without DME (16). Aflibercept produced greater DRSS improvement and reduced development of vision-threatening complications and center-involved DME compared with sham (16). Within this framework, PANORAMA introduces the question of preemptive retinal therapy in high-risk patients before PDR or CI-DME has developed.

##### 3.2.2.5 DRCR protocol W

Protocol W tested preventive aflibercept in moderate to severe NPDR without CI-DME over longer follow-up (16, 17). The trial demonstrated reduced progression to vision-threatening complications, but long-term visual acuity benefit was limited or nuanced (17). This is important for patient education because it shows that early treatment may reduce anatomic progression but must be balanced against injection burden, follow-up feasibility, and patient-specific risk.

##### 3.2.2.6 Conclusions

This stage represents an important transition point in diabetic retinopathy management, where increasing systemic metabolic burden begins to correlate with clinically meaningful retinal pathology and future risk of vision-threatening complications. Collectively, these studies demonstrate that management decisions should not rely solely on a static retinal image, but should also incorporate systemic disease severity, progression risk, reliability of follow-up, treatment burden, and patient-specific factors when determining whether closer observation or earlier retinal intervention may be appropriate (4–8, 14, 16, 17).

#### 3.2.3 Group 3A—Center-involving diabetic macular edema: OCT-guided diagnosis and treatment

##### 3.2.3.1 Goal

This section examines edema-predominant diabetic retinal disease, particularly center-involving diabetic macular edema (CI-DME), in which retinal pathology primarily affects the macula and central visual function. Emphasis is placed on OCT-driven diagnosis, disease monitoring, and treatment selection, while also supporting patient education regarding why fluid accumulation within the center of the retina substantially alters visual prognosis, treatment urgency, and long-term management strategy (15, 18–22).

##### 3.2.3.2 Diagnostic criteria

- OCT-confirmed center-involving diabetic macular edema (15, 18–22).
- Vision may be preserved or reduced; baseline visual acuity is a major treatment modifier (18–22).
- Often occurs in longstanding diabetes and may coexist with NPDR or PDR (4–8, 18–22).

##### 3.2.3.3 DRCR OCT reproducibility study

The DRCR OCT reproducibility study established central subfield thickness as a reliable quantitative monitoring endpoint in DME (15). This is critical because the DME branch of the framework depends on OCT-based diagnosis and treatment response assessment. It provides the measurement backbone for interpreting DME trials and for explaining to patients why OCT scans are repeated over time (15, 18–22).

##### 3.2.3.4 Protocol T

Protocol T compared aflibercept, bevacizumab, and ranibizumab for center-involved DME. The trial showed that all three agents improved vision, but relative effectiveness depended on baseline visual acuity, with aflibercept having greater benefit in eyes with worse baseline vision (18). This trial is central to treatment selection because it demonstrates that drug choice is not arbitrary; it depends on presenting vision, expected response, cost, and treatment context (18).

##### 3.2.3.5 VIVID/VISTA

VIVID and VISTA compared intravitreal aflibercept with macular laser for central-involved DME over extended follow-up (19). These trials established anti-VEGF therapy as superior to laser for many patients with CI-DME (19). They help explain the modern shift away from laser as first-line therapy in vision-threatening DME while preserving laser as part of historical context and selected management strategies.

##### 3.2.3.6 Protocol V

Protocol V evaluated aflibercept, laser, and observation in eyes with center-involved DME and good visual acuity (20). The trial found no significant difference in vision loss at two years among initial strategies when rescue treatment was available, supporting observation in selected patients with good vision (20). This is particularly important for patient education because it explains why some patients with swelling are monitored rather than injected immediately (20).

##### 3.2.3.7 YOSEMITE/RHINE

YOSEMITE and RHINE evaluated faricimab treat-and-extend regimens in DME and demonstrated maintained visual gains with extended dosing in many patients (21). These trials are important because they shift the discussion from efficacy alone to durability and treatment burden. For patients, they help explain why newer therapies may aim to reduce visit and injection frequency while preserving outcomes (21, 22).

##### 3.2.3.8 PHOTON

PHOTON evaluated high-dose aflibercept 8 mg at extended dosing intervals for DME (22). The trial supports the concept that higher-dose therapy may maintain visual and anatomic outcomes with fewer injections compared with standard dosing intervals in selected patients (22). It belongs in the modern durability section because treatment burden is a major practical barrier for chronic diabetic eye disease.

##### 3.2.3.9 Conclusions

Treatment decisions in CI-DME depend on OCT findings, vision, baseline severity, patient burden, and follow-up reliability. Not all edema requires immediate treatment, but anti-VEGF therapy is dominant when intervention is needed.

#### 3.2.4 Group 3B—Proliferative diabetic retinopathy: selecting between anti-VEGF and panretinal photocoagulation

##### 3.2.4.1 Goal

This section examines proliferative diabetic retinopathy (PDR), a stage of disease characterized by retinal neovascularization arising from severe ischemic and microvascular compromise. Emphasis is placed on individualized treatment selection, including panretinal photocoagulation, anti-VEGF therapy, and surgical considerations, while illustrating why management of neovascular disease is not a one-size-fits-all approach and must instead incorporate systemic disease burden, retinal severity, visual status, treatment durability, and reliability of follow-up (4–8, 23–29).

##### 3.2.4.2 Diagnostic criteria

- Neovascularization of the disc or elsewhere on examination or imaging (13, 23–26).
- May be associated with vitreous or preretinal hemorrhage (23, 27–29).
- High-risk PDR is historically defined by features associated with substantial risk of severe visual loss (23).

##### 3.2.4.3 DRS report No. 8

DRS Report No. 8 established the clinical application of photocoagulation for proliferative diabetic retinopathy and showed that photocoagulation reduced severe visual loss (23). It is the historical anchor for PRP as the standard treatment for high-risk PDR. Including DRS helps patients and clinicians understand why laser remains important even as anti-VEGF therapy has expanded treatment options (23–26).

##### 3.2.4.4 Protocol S

Protocol S compared ranibizumab with panretinal photocoagulation for PDR (24). Ranibizumab was noninferior to PRP for visual acuity outcomes at two years, with differences in DME incidence, visual field loss, and treatment burden (24, 25). This trial is essential because it shows that treatment choice depends not only on retinal findings, but also on adherence, access, cost, and ability to maintain frequent follow-up.

##### 3.2.4.5 Protocol S 5-year outcomes

The 5-year Protocol S results provide long-term durability and complication data for ranibizumab vs. PRP (25). They show that both strategies can produce favorable visual outcomes, but ongoing follow-up and treatment burden remain central to interpretation (24, 25). This supports the integrated framework because systemic reliability and access to care influence whether an injection-based strategy is practical.

##### 3.2.4.6 CLARITY

CLARITY compared aflibercept with PRP for proliferative diabetic retinopathy and demonstrated favorable one-year outcomes with aflibercept (26). It provides independent RCT support for anti-VEGF therapy as a viable treatment option in PDR (26). Together with Protocol S, CLARITY shows that PDR management has evolved beyond laser-only treatment, while still requiring individualized decision-making.

##### 3.2.4.7 Conclusions

PDR treatment requires balancing efficacy, durability, follow-up reliability, patient burden, and systemic context. Anti-VEGF and PRP are not interchangeable for every patient, even when visual outcomes are similar.

#### 3.2.5 Group 4—Advanced diabetic retinopathy: transition to procedural and surgical management

##### 3.2.5.1 Goal

This section examines advanced diabetic retinopathy complicated by vitreous hemorrhage, tractional retinal detachment, persistent neovascularization, or other vision-threatening pathology requiring procedural or surgical intervention. Emphasis is placed on the transition from medical retina management to laser therapy, intravitreal pharmacotherapy, and vitrectomy-based decision-making, while highlighting how retinal anatomy, visual potential, systemic disease burden, and reliability of follow-up influence treatment selection in advanced disease states (23–29).

##### 3.2.5.2 Diagnostic criteria

- Vitreous hemorrhage, especially non-clearing vitreous hemorrhage (23, 27–29).
- Tractional retinal detachment or combined tractional/rhegmatogenous detachment (23, 27–29).
- Severe fibrovascular proliferation or media opacity preventing adequate retinal view (23–29).

##### 3.2.5.3 DRVS

The Diabetic Retinopathy Vitrectomy Study evaluated early vs. deferred vitrectomy for severe vitreous hemorrhage (27). It remains a foundational surgical trial because it clarified when vitrectomy may improve outcomes, particularly in severe hemorrhage and advanced proliferative disease (27). In the patient roadmap, it marks the transition from injections or laser to surgery-level management.

##### 3.2.5.4 Protocol N

Protocol N evaluated ranibizumab vs. saline for vitreous hemorrhage due to PDR (28). The trial suggested limited likelihood of a major difference in vitrectomy rates by 16 weeks, while still informing the biologic role of anti-VEGF in hemorrhage management and PRP completion (28). It helps explain why some clinicians use injection-first approaches but why injection alone may not be sufficient in advanced hemorrhagic disease.

##### 3.2.5.5 Protocol AB

Protocol AB compared initial aflibercept with vitrectomy plus PRP for vitreous hemorrhage from PDR (29). This is a key modern trial because it directly compares medical-first vs. surgery-first strategies (29). It belongs at the end of the framework because it helps patients understand that advanced DR treatment may involve procedure timing, visual recovery trajectory, and individualized surgical decision-making.

##### 3.2.5.6 Evidence regarding retinal structural effects of PPV

In addition to restoring retinal anatomy and improving visual outcomes in appropriately selected patients, concerns have been raised regarding whether pars plana vitrectomy (PPV) itself may adversely affect retinal neural structures. However, emerging evidence suggests that PPV does not appear to result in greater retinal structural damage than panretinal photocoagulation (PRP) alone in eyes with severe non-proliferative diabetic retinopathy. In a recent comparative study, retinal layer thickness and structural integrity following PPV were comparable to eyes managed with PRP, suggesting that the anatomical benefits of surgery are not accompanied by additional measurable retinal structural injury. Although further prospective studies are warranted, these findings provide additional reassurance when considering surgical intervention for advanced diabetic retinopathy and further support individualized, stage-specific surgical decision-making within the proposed endocrine-retina framework (34).

##### 3.2.5.7 Conclusions

Advanced disease is procedural and timing-dependent. At this stage, the question is no longer simply which injection or laser, but whether medical-first or surgery-first management is most appropriate for the patient.

#### 3.2.6 From screening to staging: diagnostic tools for diabetic retinopathy

##### 3.2.6.1 Goal

This section defines how patients transition from endocrine classification and systemic metabolic risk assessment into retinal diagnosis, staging, and longitudinal ophthalmic monitoring. Emphasis is placed on screening modalities, diagnostic imaging, and severity classification systems used to identify and stratify diabetic retinopathy, while maintaining separation between diagnostic frameworks and treatment-effect evidence so that screening tools, staging systems, and therapeutic trials remain conceptually distinct within the overall endocrine-retina model (11–15).

##### 3.2.6.2 ETDRS report no. 10

ETDRS Report No. 10 provides the grading framework for diabetic retinopathy severity from stereoscopic color fundus photographs (13). It is essential because DRSS change is a shared endpoint across many modern trials (13, 26). In this review, ETDRS serves as the ophthalmic language that allows endocrine-defined patient risk to be mapped onto retinal severity (4–8, 13).

##### 3.2.6.3 AI screening studies

Abràmoff and EyeArt studies support scalable detection of diabetic retinopathy in non-specialty settings (11, 12). They belong in the diagnostic framework rather than treatment evidence because they help determine when an endocrine patient should enter ophthalmic care. Their patient-facing implication is straightforward: diabetic eye disease can be detected before symptoms, and screening can occur outside the retina specialist clinic in validated programs (11, 12).

##### 3.2.6.4 Ultrawide-field imaging

Silva and related ultrawide-field imaging studies demonstrated that predominantly peripheral retinal lesions identified outside traditional ETDRS imaging fields are associated with increased risk of diabetic retinopathy progression (14). This supports more sensitive risk stratification for selected patients. It also aligns with the review motivation that older or narrower screening approaches may miss clinically meaningful disease patterns (14).

##### 3.2.6.5 OCT for DME

OCT provides the quantitative basis for identifying center-involving macular edema and monitoring response to therapy (15, 18–22). The DRCR reproducibility work supports using central subfield thickness as a reliable outcome measure (15). For patients, OCT can be explained as the scan that shows whether diabetes is causing swelling in the part of the retina used for sharp central vision.

##### 3.2.6.6 Functional assessment

Although structural imaging remains the foundation of diabetic retinopathy screening and staging, growing evidence suggests that functional retinal abnormalities may precede clinically detectable structural changes. Electrophysiologic techniques, including periodic-stimulus electroretinography (ERG), can detect subtle alterations in retinal function that reflect early neurovascular dysfunction before overt vascular lesions become apparent. While these approaches are not currently part of routine diabetic retinopathy screening, they provide valuable insight into the functional consequences of early retinal injury and may serve as complementary biomarkers for future risk stratification and disease monitoring as electrophysiologic technologies continue to evolve (36).

Recent advances in artificial intelligence further expand the role of retinal imaging beyond diabetic retinopathy detection alone. A large multicenter study by Zhang et al. developed a multitask AI framework (Reti-Pioneer) capable of screening for multiple endocrine and metabolic disorders—including type 2 diabetes, hypertension, hyperlipidemia, gout, osteoporosis, and thyroid disease—from a single color fundus photograph. Developed using more than 107,000 retinal images and validated across multiple external cohorts, the framework demonstrated robust diagnostic performance while completing image analysis in approximately 30 s during prospective primary care testing. These findings illustrate the growing potential of retinal imaging as a noninvasive platform for systemic disease screening and provide compelling support for the endocrine-retina framework proposed in this review, in which retinal assessment serves as a bridge between ophthalmology and systemic metabolic health (35).

##### 3.2.6.7 Conclusions

Collectively, these diagnostic and staging studies demonstrate that diabetic retinopathy assessment depends on more than the presence or absence of visible retinal lesions alone. Modern screening and imaging modalities now allow clinicians to identify retinal disease earlier, stratify progression risk more precisely, and monitor treatment response quantitatively across the disease continuum (11–15). Within the endocrine-retina framework, these tools serve as the bridge between systemic metabolic risk and clinically actionable ophthalmic management decisions.

#### 3.2.7 Framework for quantitative synthesis and future meta-analysis

The proposed endocrine-retina framework organizes diabetic retinopathy literature according to systemic metabolic disease stage, retinal phenotype, diagnostic modality, and treatment context rather than treating diabetic retinopathy as a single homogeneous entity (1–30). Within this model, patients are first categorized according to endocrine-defined systemic risk and disease burden, then mapped onto retinal staging, imaging findings, and stage-specific ophthalmologic interventions (1–30). This structure allows clinically related studies to be grouped together while preserving important distinctions between screening, prevention, medical retina management, proliferative disease, and surgical-level complications.

The framework is designed to support future quantitative synthesis where pooling is methodologically appropriate. Because disease stages, imaging endpoints, and interventions differ substantially across diabetic retinopathy populations, a single pooled meta-analysis across all studies would not be scientifically appropriate. Instead, endpoints should be pooled within clinically coherent groups defined by retinal phenotype, treatment strategy, and systemic disease context (13–29).

##### 3.2.7.1 Core endpoints

- DRSS regression or progression, including two-step or greater change when available (13, 16–26).
- Incidence of center-involving diabetic macular edema (16–22).
- Incidence or progression to proliferative diabetic retinopathy (16, 17, 23–26).
- Need for laser therapy, including focal/grid laser or panretinal photocoagulation (7, 19, 20, 23–29).
- Need for vitrectomy or other surgical intervention (27–29).
- Best-corrected visual acuity change, typically ETDRS letters (18–29).
- OCT central subfield thickness or central retinal thickness (15, 18–22).
- Injection frequency, visit burden, and treatment durability (21, 22, 24–26).

##### 3.2.7.2 Likely meta-analysis groupings

- Systemic risk modification and retinal outcomes: UKPDS, ACCORD Eye, FIELD, and DPP/DPPOS would likely function primarily as a structured narrative and feasibility-analysis group because interventions, populations, and endocrine treatment strategies differ substantially across studies (3, 5–7).
- NPDR prophylaxis and early intervention: PANORAMA and Protocol W represent a clinically coherent grouping focused on prevention of progression in moderate-to-severe NPDR, including DRSS improvement, CI-DME incidence, progression to PDR, and treatment burden outcomes (16, 17).
- CI-DME treatment strategies: Protocol T, VIVID/VISTA, Protocol V, YOSEMITE/RHINE, and PHOTON may be grouped according to comparable treatment questions involving anti-VEGF selection, durability, observation vs. intervention, and OCT-based edema outcomes rather than pooled indiscriminately as a single therapeutic category (18–22).
- PDR treatment strategy comparisons: Protocol S and CLARITY represent clinically comparable studies evaluating anti-VEGF therapy vs. panretinal photocoagulation for proliferative diabetic retinopathy, with DRS serving primarily as the historical treatment anchor rather than a directly pooled comparator study (23–26).
- Advanced complications and surgical-level disease: DRVS, Protocol N, and Protocol AB are likely best suited for structured narrative synthesis because surgical techniques, anti-VEGF use patterns, and treatment eras differ substantially across studies evaluating vitreous hemorrhage and advanced proliferative disease (27–29).

## 4 Synthesis and discussion

This review presents an integrated approach to diabetic retinopathy that places systemic metabolic disease at the center of clinical decision-making. By synthesizing evidence from endocrinology and ophthalmology, it follows the natural progression of diabetes from metabolic dysfunction to retinal pathology, emphasizing how systemic risk factors influence retinal phenotype, diagnostic evaluation, therapeutic selection, and patient education (1, 4–10, 13–29). This perspective highlights diabetic retinopathy as a dynamic manifestation of systemic disease and reinforces the value of coordinated care across medical specialties (3–10, 13–29). Rather than introducing new clinical recommendations, the review organizes existing evidence into a practical model that facilitates interdisciplinary management of diabetic retinopathy (1, 31, 32).

First, the risk of retinopathy is determined by cumulative metabolic exposure, often preceding clinically detectable retinal changes (3–10). Recognition of inflammatory and neurodegenerative mechanisms further reinforces this concept by suggesting that retinal injury may begin before clinically detectable vascular abnormalities develop, emphasizing the importance of early systemic metabolic intervention (10, 33). This supports prioritizing early systemic intervention and highlights the limitations of relying solely on cross-sectional retinal imaging for risk assessment (4–10, 14).

Second, similar retinal phenotypes may arise from heterogeneous systemic conditions. Patients with the same retinal stage may differ substantially in diabetes duration, A1C trajectory, renal disease, blood pressure control, lipid profile, and overall complication burden (4–8). As a result, disease progression and treatment response cannot be fully predicted by retinal findings alone (4–10, 30).

Third, treatment effectiveness and treatment appropriateness are context-dependent (16–29). Randomized trials establish the efficacy of anti-VEGF therapy, PRP, laser, and vitrectomy in specific retinal disease states, but real-world application must also account for adherence, access to care, systemic control, transportation barriers, medication cost, and ability to attend repeated appointments (21, 22, 24, 25, 29).

Fourth, screening technologies and endocrine care pathways create an opportunity to identify patients earlier (11, 12, 14). AI-based screening, primary-care workflows, and validated imaging programs may reduce missed screening and allow earlier ophthalmic referral (11, 12). This is particularly important for patients in rural or underserved settings, where delays in specialty access may contribute to preventable vision loss (4–8, 11, 12). Recent advances in artificial intelligence further reinforce this interdisciplinary model. A large multicenter AI framework demonstrated that a single retinal photograph can identify multiple endocrine and metabolic disorders in addition to diabetic retinopathy, illustrating the expanding role of retinal imaging as a noninvasive biomarker of systemic health. These findings provide compelling support for the proposed endocrine-retina framework by demonstrating how ophthalmic imaging can facilitate earlier detection of systemic disease while strengthening collaboration between ophthalmology, endocrinology, and primary care (35).

Current ADA and AAO guidelines remain the foundation for evidence-based screening, diagnosis, and management of diabetic retinopathy (1, 32). The framework presented here complements these recommendations by integrating systemic metabolic disease with retinal findings across the continuum of diabetes, reinforcing interdisciplinary communication and supporting individualized clinical decision-making and patient education (4–10, 13–30).

Finally, emerging evidence supporting heterogeneity in diabetes phenotypes suggests that retinal disease progression cannot be fully understood without considering underlying metabolic subtype and systemic disease burden (4–10, 30). Applying endocrinology as a lens does not replace retinal diagnosis; rather, it enriches interpretation of retinal findings and may improve individualized management (9, 30).

These observations also identify several important directions for future clinical research. Prospective studies integrating endocrine phenotyping with retinal imaging may help determine whether distinct metabolic subtypes predict differential rates of DR progression, DME development, or response to anti-VEGF therapy (9, 30). Future work should also evaluate whether systemic biomarkers such as renal function, albuminuria, inflammatory markers, or longitudinal glycemic variability can improve individualized retinal risk prediction beyond traditional staging systems alone (4–10). In addition, emerging screening technologies, including autonomous AI systems and ultrawide-field imaging, warrant further study in rural and underserved populations to determine whether earlier detection meaningfully reduces vision-threatening complications and disparities in access to care (11, 12, 14). Finally, future comparative-effectiveness studies examining durability-focused treatment paradigms, adherence patterns, and real-world barriers to follow-up may help refine individualized management strategies across the full spectrum of diabetic retinopathy severity (21, 22, 24–29).

The synthesis of these lines of evidence supports a shift toward a more integrated, patient-centered model of care. Such a model can improve clinical consistency, enhance communication between specialties, and provide a clearer framework for patient education. Taken together, these findings suggest that current retina-centric staging systems may incompletely capture disease risk without integration of longitudinal systemic data, highlighting the need for a more unified, patient-centered model of care.

Integration may be accomplished through closer alignment of endocrinology, primary care, and ophthalmology workflows, including shared risk stratification models that incorporate systemic metabolic variables alongside retinal imaging findings (4–10, 13–15). Earlier referral pathways using validated AI-based screening systems and ultrawide-field imaging may allow endocrine-defined high-risk patients to enter ophthalmic care before vision-threatening complications develop (11, 12, 14). Standardized communication of retinal severity, systemic disease burden, treatment burden, and follow-up reliability across specialties may also improve coordination of care and facilitate earlier intervention in patients at highest risk for progression (4–8, 16–29).

Patient-centered strategies may be strengthened through development of longitudinal educational tools that explain diabetic retinopathy as part of systemic diabetes progression rather than as an isolated eye disease. Framing disease stages according to understandable clinical transitions — such as screening, early retinal change, edema development, neovascular disease, and surgical-level complications — may help patients better understand why treatment recommendations evolve over time (13, 16–29). Similarly, incorporating individualized discussions regarding injection burden, transportation limitations, appointment frequency, financial barriers, and systemic disease control may improve adherence, shared decision-making, and long-term engagement with diabetic eye care (21, 22, 24–29).

## 5 A patient roadmap for understanding diabetic retinopathy

A central motivation for this review is the development of a concise patient education resource that translates the proposed endocrine-retina framework into practical language for patients and caregivers. Because many patients struggle to understand how systemic diabetes influences retinal disease, the accompanying supplementary pamphlet summarizes key concepts regarding disease progression, screening, treatment options, and communication with both ophthalmology and diabetes care teams.

The proposed roadmap provides five core messages:

- Diabetic retinopathy begins as a complication of systemic diabetes, not as an isolated eye problem.
- Risk is influenced by A1C, duration of diabetes, blood pressure, kidney disease, cholesterol, and follow-up adherence.
- Screening is necessary even when vision is normal, because early DR may be asymptomatic.
- Treatment options depend on retinal findings and may include monitoring, injections, laser therapy, or surgery. Laser treatment remains important in selected patients, particularly in proliferative diabetic retinopathy, even as anti-VEGF therapy has expanded modern treatment options (19, 23–29).
- Patients can reduce risk by improving metabolic control, attending regular eye examinations, and maintaining follow-up adherence. Evidence from major clinical trials demonstrates that glycemic control, systemic risk-factor management, and timely ophthalmic monitoring are associated with reduced progression of diabetic retinopathy and vision-threatening complications (3–7, 16, 17).

By presenting DR as a continuum rather than a series of isolated events, a patient pamphlet based on this framework may reduce confusion, improve adherence, and help patients anticipate future care decisions. The accompanying supplementary patient pamphlet expands these concepts into a practical educational resource that includes stage-specific management, questions patients can discuss with their healthcare team, and tools to support shared decision-making.
