# Supplementary material for: Endocrinology concepts for diabetic retinopathy care: bridging systemic metabolic control with retinal disease management and patient education
Source: Front Med (Lausanne). 2026 Aug 28;13:1904880. doi: 10.3389/fmed.2026.1904880 (PMC13563000; doi:10.3389/fmed.2026.1904880)
Supplement: Supplementary file 1 [file Supplementaryfile1.pdf]

Supplementary Material

1 Supplementary Data

2 Appendix: One-Page Patient Pamphlet Text

2.1 Diabetic Retinopathy: What You Need to Know

2.1.1 Diabetic Retinopathy: Protecting Your Vision

You have more power than you think. Diabetic retinopathy is damage to the small blood vessels in the **retina** (the light-sensitive layer at the back of your eye). Diabetic retinopathy reflects how well diabetes has been controlled over time—it is not just an eye problem, but part of the overall disease. The good news: early detection and good diabetes care can prevent vision loss in many people.

2.1.1.1 Why Screening Matters — Even If You See Fine

- Early diabetic retinopathy usually has **no symptoms**.
- Waiting for blurry vision or floaters often means the disease is more advanced.
- **Regular annual eye exams** can catch problems early — when they are easiest to treat. Depending on severity, more frequent exams may be necessary.

2.1.1.2 What Increases Your Risk?

- Longer time living with diabetes (especially 5–10+ years)
- Higher A1C (a 3 month snapshot of your average blood sugar levels)
- High blood pressure
- Kidney disease or protein in urine (albuminuria)
- High cholesterol or triglycerides
- Smoking
- Missing regular eye exams

Good news: Improving any of these factors can slow or reduce damage.

2.1.1.3 How Diabetic Retinopathy Typically Progresses

Stage	What's Happening	Common Symptoms	Usual Approach
**Early / Mild**	Small changes in retinal blood vessels	Usually none	Good diabetes control + yearly screening

Stage	What's Happening	Common Symptoms	Usual Approach
**Moderate**	More vessel damage	Usually none	Closer monitoring (every 6–12 months)
**Macular Edema**	Swelling in the central retina	Blurry central vision	Observation or eye injections
**Proliferative (PDR)**	Abnormal new blood vessels grow	Floaters, sudden vision loss	Injections and/or laser
**Advanced**	Bleeding or scar tissue in the eye	Severe vision loss possible	Injections, laser, or surgery

2.1.1.4 Modern Treatment Options

- **Monitoring –** Many people stay stable with good control and regular checks.
- **Anti-VEGF Injections –** Medications injected into the eye that reduce swelling and stop abnormal blood vessels (most common treatment today).
- **Laser Treatment** – Helps seal leaking vessels or prevent bleeding (still very useful in certain cases).
- **Surgery (Vitrectomy) –** Used for severe bleeding or scar tissue.

Treatment is highly individualized. Your retina specialist will choose the best option based on your eye findings, vision, and overall health.

2.1.1.5 What You Can Do Right Now

1. Work with both your endocrinologist/primary care doctor and your eye doctor to reach your A1C, blood pressure, and cholesterol goals.
2. Get a dilated eye exam at least once a year — or more often if you already have diabetic retinopathy..
3. Take your diabetes and eye medications as prescribed, and make sure each doctor knows about changes in your treatment plan.
4. Eat well, stay active, and avoid smoking to help protect both your overall health and your vision.
5. Keep all eye appointments and follow-up visits — consistent monitoring helps your care team catch problems early.
6. Report new symptoms immediately, including sudden floaters, flashes, dark spots, blurred vision, or vision loss.
7. Think of your diabetes care and eye care as connected: good communication between your endocrinologist and ophthalmologist can help prevent vision-threatening complications before they become severe.

2.1.1.6 When should I call my eye doctor right away?

- Sudden blurry or distorted vision
- A sudden increase in floaters
- Flashes of light
- A dark curtain or shadow in your vision

- Sudden vision loss in one or both eyes

These symptoms do not always mean your retinopathy has worsened, but they should be evaluated as soon as possible.

2.1.1.7 Questions to Ask Your Doctors

2.1.2 Questions to Ask Your Eye Doctor (Ophthalmologist/Retina Specialist)

• What stage is my diabetic retinopathy, and what does that mean for my future vision risk?
• Is there swelling (macular edema) in the center of my retina?
• Do I need treatment now, or is monitoring the best option?
• Would laser treatment, injections, or surgery ever become necessary for my condition?
• How often should I follow up for eye exams or retinal imaging?
• Are there newer treatment options that may reduce the number of injections or visits I need?

• Is my diabetic retinopathy improving, stable, or getting worse compared with my last visit?

2.1.3 Questions to Ask Your Endocrinologist or Primary Diabetes Doctor

• How well are my A1C, blood pressure, cholesterol, and kidney function being controlled?
• Which of my diabetes risk factors may be increasing my risk for vision loss?
• Are there changes to my medications or lifestyle plan that could better protect my eyes?
• How can my diabetes care team and eye doctor better coordinate my treatment plan?
• Should I be screened more often based on my current diabetes control or complication burden?

• What A1C goal is appropriate for me, and how does it affect my eye health?

2.1.4 Questions to Help Connect Both Parts of Your Care

• How does my overall diabetes control relate to what my eye doctor is seeing in my retina?
• Are my retinal findings getting better, worse, or staying stable over time?
• What signs or symptoms should prompt me to contact my doctors immediately?
• What can I do now to reduce my risk of needing injections, laser treatment, or surgery in the future?

• Does my eye doctor need any information from my diabetes care team before my next visit?

2.2 "Know Your Numbers"

Know Your Numbers and try to make improvements by your next appointment

Goal measurements

- Goal A1C: _______
- Goal Blood Pressure: _______
- Goal Cholesterol: _______

Most Recent measurements

- Latest A1C: _______
- Latest Blood Pressure: _______
- Latest Cholesterol: _______
- Last Eye Exam: _______
- Next Eye Exam: _______

2.2.1 Remember

Good vision depends on both healthy eyes and healthy diabetes management. Your endocrinologist, primary care physician, and ophthalmologist each play an important role in protecting your sight.

2.3 Additional Resources

• American Diabetes Association (ADA) — diabetes standards, prevention, and patient education
• National Eye Institute (NEI) — diabetic retinopathy education and vision resources
• American Academy of Ophthalmology (AAO) — retinal disease and treatment information

---

Main Message Diabetic retinopathy is a complication of diabetes — not an inevitable result. With good systemic control, regular screening, and timely eye care, most people with diabetes keep good vision for life.

Take this pamphlet to your next appointment. Share it with family members who help with your diabetes care.

Created as part of an integrated endocrinology–ophthalmology review framework. Talk to your healthcare team for personalized advice.
